# MNE-BIDS: Organizing electrophysiological data into the BIDS format and facilitating their analysis

**DOI:** 10.21105/joss.01896

**Published:** 2019-12-18

**Authors:** Stefan Appelhoff, Matthew Sanderson, Teon L Brooks, Marijn van Vliet, Romain Quentin, Chris Holdgraf, Maximilien Chaumon, Ezequiel Mikulan, Kambiz Tavabi, Richard Höchenberger, Dominik Welke, Clemens Brunner, Alexander P Rockhill, Eric Larson, Alexandre Gramfort, Mainak Jas

**Affiliations:** 1 Center for Adaptive Rationality, Max Planck Institute for Human Development, Berlin, Germany; 2 Department of Cognitive Sciences, Macquarie University, Sydney, Australia; 3 Mozilla; 4 Department of Neuroscience and Biomedical Engineering, Aalto University, Espoo, Finland; 5 Human Cortical Physiology and Neurorehabilitation Section, NINDS, NIH, Bethesda, Maryland 20892; 6 UC Berkeley, Project Jupyter; 7 Institut du cerveau et de la moelle épinière (ICM), Paris, France; 8 Department of Biomedical and Clinical Sciences ‘L. Sacco’, University of Milan, Milan, Italy; 9 Institute for Learning and Brain Sciences, University of Washington, Seattle, WA, USA; 10 Institute of Neuroscience and Medicine (INM-3), Research Center Jülich, Germany; 11 Max-Planck-Institute for Empirical Aesthetics, Frankfurt a.M., Germany; 12 Institute of Psychology, University of Graz, Austria; 13 University of Oregon, Eugene OR, USA; 14 Université Paris-Saclay, Inria, CEA, Palaiseau, France; 15 Athinoula A. Martinos Center for Biomedical Imaging, Massachusetts General Hospital, Charlestown, MA, USA

## Abstract

The development of the Brain Imaging Data Structure (BIDS; Gorgolewski et al., 2016) gave the neuroscientific community a standard to organize and share data. BIDS prescribes file naming conventions and a folder structure to store data in a set of already existing file formats. Next to rules about organization of the data itself, BIDS provides standardized templates to store associated metadata in the form of Javascript Object Notation (JSON) and tab separated value (TSV) files. It thus facilitates data sharing, eases metadata querying, and enables automatic data analysis pipelines. BIDS is a rich system to curate, aggregate, and annotate neuroimaging databases.

While BIDS was originally intended for magnetic resonance imaging (MRI) data, it has extensions for other data modalities including: magnetoencephalography (MEG; Niso et al., 2018), electroencephalography (EEG; Pernet et al., 2019), and intracranial encephalography (iEEG; Holdgraf et al., 2019). Software packages analyzing MEG, EEG, and iEEG are now starting to support data organized using the BIDS standard, thereby becoming “BIDS compatible”. Within the Python ecosystem, MNE–Python (Gramfort et al., 2013) is a major software package for electrophysiology data analysis, and extending its functionality to support BIDS would be a great benefit for its growing user base. For this reason, we developed a dedicated Python software package *MNE–BIDS with the goal of providing a programmable interface for BIDS datasets in electrophysiology with MNE-Python.* MNE–BIDS allows users to re-organize data into BIDS formats, store associated metadata after anonymization, extract information necessary for preprocessing, and read the data into MNE–Python objects, ready for source localization.

Starting with a single directory full of data files with arbitrary names, MNE–BIDS can be used to extract existing metadata, reorganize the files into the BIDS format, and write additional metadata. All the conversion routines are thoroughly tested by running the output through the BIDS validator. Moreover, MNE–BIDS supports converting data formats that are not BIDS compatible into permissible formats. These utilities allow users to easily convert their datasets to BIDS in a matter of minutes, rather than hours of manual labour.

In addition to this core functionality, MNE–BIDS is continuously being extended to facilitate the analysis of BIDS formatted data. Some features include: reading a BIDS dataset as a set of Python objects for analysis with MNE–Python, defacing T1-weighted anatomical MRI images to anonymize data and facilitate sharing, and saving anatomical landmark coordinates to enable coregistration between the MEG/EEG and MRI data, which is necessary for computation of forward and inverse solutions.

Users can easily install MNE–BIDS on all major platforms via pip and conda, and its functionality is continuously tested on Windows, macOS, and Linux. Other than the core dependencies for scientific computing (numpy, scipy) and handling of MEG/EEG data (mne), MNE–BIDS has minimal dependencies, all of which are optional. The Application Programming Interface (API) of the package is stable and extensively documented and explained in examples (https://mne.tools/mne-bids/). In addition, a command-line interface is provided that allows non-Python users to benefit from the core functionality.

As of writing, MNE–BIDS has received code contributions from 15 contributors and its user base is steadily growing. Code development is active and the developer team is committed to provide timely support for issues opened on the GitHub issue tracker.

MNE–BIDS is used as a dependency in several other software packages such as the MNE-study-template, an automated pipeline for group analysis with MNE (Jas et al., 2018), and Biscuit, a graphical user interface to format BIDS data. Lastly, several large institutions have adopted MNE–BIDS for their workflows such as the Martinos Center for Biomedical Imaging.

The developer team is excited to improve the state of the art in data handling and looking forward to welcoming new contributors and users.
